# Statistical Coding and Decoding of Heartbeat Intervals

**DOI:** 10.1371/journal.pone.0020227

**Published:** 2011-06-09

**Authors:** Fausto Lucena, Allan Kardec Barros, José C. Príncipe, Noboru Ohnishi

**Affiliations:** 1 Biological Information Engineering Laboratory, Nagoya University, Nagoya, Aichi, Japan; 2 Laboratorio para Processamento da Informação Biológica, Universidade Federal do Maranhão, São Luís, Maranhão, Brazil; 3 National Agency of Petroleum, Gas and Biofuels, Rio de Janeiro, Brazil; 4 Department of Electrical and Computer Engineering, University of Florida, Gainesville, Florida, United States of America; Mount Sinai School of Medicine, United States of America

## Abstract

The heart integrates neuroregulatory messages into specific bands of frequency, such that the overall amplitude spectrum of the cardiac output reflects the variations of the autonomic nervous system. This modulatory mechanism seems to be well adjusted to the unpredictability of the cardiac demand, maintaining a proper cardiac regulation. A longstanding theory holds that biological organisms facing an ever-changing environment are likely to evolve adaptive mechanisms to extract essential features in order to adjust their behavior. The key question, however, has been to understand how the neural circuitry self-organizes these feature detectors to select behaviorally relevant information. Previous studies in computational perception suggest that a neural population enhances information that is important for survival by minimizing the statistical redundancy of the stimuli. Herein we investigate whether the cardiac system makes use of a redundancy reduction strategy to regulate the cardiac rhythm. Based on a network of neural filters optimized to code heartbeat intervals, we learn a population code that maximizes the information across the neural ensemble. The emerging population code displays filter tuning proprieties whose characteristics explain diverse aspects of the autonomic cardiac regulation, such as the compromise between fast and slow cardiac responses. We show that the filters yield responses that are quantitatively similar to observed heart rate responses during direct sympathetic or parasympathetic nerve stimulation. Our findings suggest that the heart decodes autonomic stimuli according to information theory principles analogous to how perceptual cues are encoded by sensory systems.

## Introduction

In order to explain a biological information processing strategy, one must understand how the stimuli is encoded and decoded along the different neuronal pathways [1]–[4]. Adaptation represents one of the most important innate characteristics of the nervous system. For example, visual and auditory sensory systems adopt different mechanisms through evolutionary processes to achieve the same general sparsity encoding strategy [5]–[8]. Curiously, the reflexive nervous system exhibits an astonishing capacity in adjusting autonomic functions to a variety of internal and external behavioral conditions. It is likely that the neural processing of regulatory messages evolved to make the best use of the incoming information to ensure autonomic function accuracy. It remains unclear, however, whether the autonomic nervous system shares guiding principles analogous to sensory systems. Due to the dynamic nature of the cardiac system, one needs to frame the problem in a dynamical system setting, exploring time for the computation.

In the cardiac system, adaptation of the heart rhythm is mediated by the neuroregulatory messages sent by the autonomous nervous system. Physiologically, the electrical stimuli arriving in the heart from reflexive pathways are expressed by beat-to-beat variations assumed to reflect the interplay between the sympathetic and the parasympathetic activities [9]. Under normal conditions, the cardiac rhythm depends on the mutual entrainment of pacemaker cells in the sinoatrial (SA) node. At the system's level, pacemaker cells synchronize their intrinsic frequency on the neuroregulatory stimuli to which they are exposed [10]. If the heart processes neuroregulatory messages based on biological constraints, one would expect a strategy to adapt the information flow to maintain a proper regulation of the cardiac rhythm.

In a dynamical setting, the simplest way to represent a complex system is to describe its input-output mapping by a filter using a reduced mathematical descriptor called the transfer function. Although the idea of filters in cardiac physiology control context might seem far fetched, transfer functions have been largely explored to study the behavior of cardiac autonomic function [11], [12]. Therefore a filter representation is useful to describe the responses of the heart using a limited number of mathematical descriptors.

According to experimental studies, the cardiac autonomic signals are modulated into different ranges of frequency [13]. There is evidence that the heart translates sympathetic and vagal stimuli compatible with low-pass filtering, differing on their corner frequencies [11], [12]. In the presence of noise and uncertainty, the cardiac system adds robustness to the cardiac rhythm through parallel built-in redundancies to avoid heart failure [14]. A bank of filters optimized to recognize self-similar patterns seems an efficient manner to maintain the cardiac regulation because it allows for independent regulation across the frequency spectrum. The challenge is to come up with a generative model that provides the characteristics of those filters just by observing the heart rhythm itself. The current experimental analysis of autonomic responses is able to derive the filtering proprieties of the heart. However, the coding mechanisms of an intact cardiac system remain unclear [15]. Despite the effort to describe the response proprieties of sinoatrial (SA) node, the autonomic cardiac regulation has not been fully understood. Previous studies are often restricted to transfer function relationships, limiting these analyses to the amplitude spectrum information.

A more principled alternative uses the statistical proprieties of heartbeat intervals to model the cardiac neuroregulatory encoding messages. Indeed, several authors [16]–[18] have argued that a more complete model characterization of the heart beat dynamics depends on the generative mechanism underlying the cardiac rhythm. In a number of recent papers [16]–[18], neural and hormonal variables (i.e., nonlinearities and nonstationarities) have been probabilistically integrated into previous cardiac models, showing that a heartbeat design system can, in part, successfully estimate the cardiac rhythm using these parameters. Whilst these models have greatly advanced our capacity of predict cardiac dynamics, they do not explain the strategy underlying the transformation of neuroregulatory commands into the cardiac rhythm.

A longstanding computational hypothesis in perception suggests that specialized neurons reduce the redundancy of messages that are frequently happening [19], [20], efficiently coding information under band limited conditions [21]. This hypothesis postulates that neurons are similar to a large set of encoders statistically independent of each other [22]. Specifically, efficient codes learned from natural images and sounds helped to clarify the functional organization and topological structure of early visual and acoustic sensory stages, respectively [5], [6]. This approach provided evidence that early neural sensory processing is strongly biased towards the behavioral importance of the stimuli [23]. This reasoning also suggests that olfactory neurons obey the same general principles [7]. But, can similar ideas be applied to uncover the neural processing of non-sensory systems, such as the cardiac system?

Herein we use a theoretical framework to investigate whether the SA node in the heart encodes neuroregulatory messages with a strategy similar to sensory systems, but of course adapted to the dynamical nature of cardiac rhythms. Using independent component analysis (ICA) with new assumptions plausible for the heart physiology, we maximize the (non-Gaussian) information in the heartbeat intervals derived from normal sinus rhythm in healthy volunteers. The filter properties emerging from the population code suggest that the efficient coding hypothesis is not limited to sensory systems as it was first assumed [20]. Rather, our analysis lends support to the idea that redundancy reduction represents a underlying strategy for functional self-organization in biological systems.

### The Relevance of Efficient Coding in Heartbeat Intervals

The concept of an efficient code [20] implicitly means that message encoding or transmission of information in a system must approach theoretical limits [24]. When this concept was first applied to computational perception by Attneave and Barlow [19], [20], one drawback was the stimulus neutrality with respect to efficient coding [23]. Thus, an important step in estimating efficient codes is to infer which stimuli convey more information to the system. Follow up research [23], [25] has shown that sensory neurons in visual or auditory cortices have preference for naturalistic signals over white-noise sources. It implies that the efficiency of the code varies from stimuli to stimuli, depending on their behavioral relevance.

In the case of the heart, this reasoning implies that reflexive stimuli are likely to cause the most efficient codes, which is a hypothesis that in principle can be experimentally verified in the laboratory. Yet, the cardiac system response is very likely altered with the current invasive procedures required to acquire such stimuli. Alternatively, it is possible to estimate a population code based on efficient coding theory using heartbeat intervals. Heartbeat intervals are signals derived from electrocardiograms that accounts for the neuroautonomic fluctuations in the heart [26]. They are obtained from the temporal difference of consecutive R-peak waves. Their temporal structure is composed of repetitive responses originated in the SA node and modulated by sympathetic and parasympathetic stimuli within the limited dynamic range of the heart.

According to previous work [27], heartbeat intervals are encoded in the spontaneous discharge of thalamic somatosensory neurons. Short-term spectral analysis of heartbeat intervals are divided in three frequency bands located at very-low (VLF) (0.00–0.03 Hz), low (LF) (0.03–0.15 Hz), and high (HF) (0.15–0.5 Hz) frequencies [9]. Several authors [28] have associated the LF band with sympathetic and parasympathetic effects due to situations in which both autonomic tones drive similar responses. For instance, heart rate variability at rest virtually disappears with vagal blockade, suggesting that low frequency variability might be related to both sympathetic and parasympathetic effects. However, in nearly all of the physiological conditions, the activation of one autonomic tone is likely to result in an inhibition of the other [26], [29]–[31]. The HF band has been associated to vagal tones and with respiratory sinus rhythm (SNA) synchronization. The VLF band is harder to estimate accurately since it requires very long windows for good estimation according to the time frequency uncertainty relation. This suggests that the heart possesses different decoding mechanisms. If so, what sorts of features are likely to emerge from a set of filters optimized to decode neuroregulatory messages using an efficient coding framework?

## Results

### The Model

**Figure 1:**
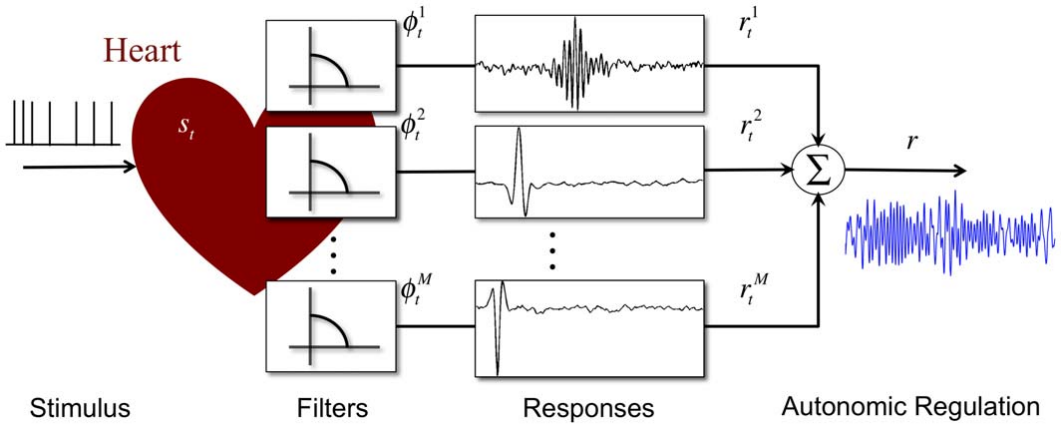
Proposed theoretical model of autonomic cardiac regulation. The cardiac response 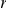 is modulated by autonomic functions stimulating the heart with stimuli of intensity 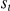 evoking a response that is represented by the output of a set of 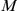 filters 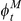.

Our generative model of the autonomic cardiac regulation (**Fig. 1**) assumes that the heart efficiently transforms an array of 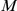 neuroregulatory impulsive messages 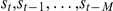 occurring at unknown times into cardiac dynamic responses 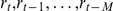 using a set of switching linear decoding filters 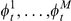 of order 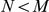, i.e. 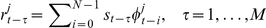 which produce the observable heart's autonomic response through an additive operation 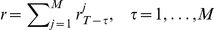.

According to this model, the goal of cardiac neural processing can be described as a way to maintain an optimal representation of the neuroregulatory information, even in presence of noise or redundancy. Note, however, that the uncertainty caused by the noise plays a fundamental role in determining which information should be encoded or removed, even deciding if redundancy should be at all used [2], [32], [33]. The aim is to maximize the information embedded in the cardiac responses reducing the redundant neurocontrol signals that may arrive.

In this efficient design, a set of encoding filters can be estimated through an iterative process of optimization that has been called independent component analysis (ICA) [34]. This method [35] searches for features-like filters (or basis functions) that transform an observed dataset into a set of elements, whose components are considered independent. Therefore the neuroregulatory components and the filter coefficients can be recoverable from the system output, i.e. after the sum. Or equivalently, a message 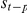 in an observation window of size 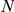 is efficiently encoded (in the sense described below) by a filter 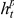 as
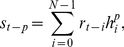
(1)


where 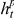 corresponds to the inverse of 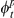.

In order to conduct this analysis, a set of hypothesis is necessary. We assume that the neuroregulatory messages are impulsive, i.e. they exist at a time scale of milliseconds (action potentials), while the filters' responses exist at the time scale of tens of seconds. Because of the fast on-off nature of the neuroregulatory messages compared with the filter responses, we assume that they can be modelled as a point process of non-overlapping Dirac delta functions during the observation window, making them statistical independent. Therefore, the filter responses can be considered impulse responses and are independent because they are the convolution of the point process with unknown filters. The goal of the analysis is to find the unknown filter coefficients by observing several windows of length 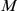 samples of the heart's autonomic response 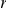 represented by heartbeat intervals. Herein, we selected heartbeat interval windows composed of 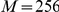 samples obtained from a set of normal sinus rhythm volunteers (see methods), to estimate with ICA the filter coefficients and the occurrence of the neuroregulatory activity. Although it is possible to derive codes with smaller windows size, this number of samples was chosen to provide sufficient information about the interactions underlying the autonomic cardiac regulation. Longer windows may invalidate the assumption of statistical independence required for the ICA decomposition because each filter may be excited by more than one neuroregulatory message.

### Decoding Population

**Figure 2:**
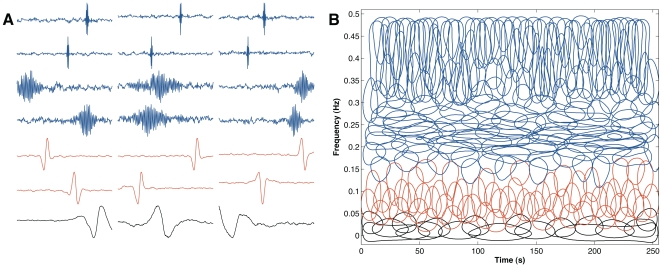
Population code optimized through heartbeat intervals derived from normal sinus rhythm volunteers. Each waveform was adapted upon a time window composed of 256 beat-intervals. (A) From a total number of 256, the plot illustrates a typical set of decoding filters organized from the highest to the lowest center frequency. Although the self-organization of the decoding population is not homogenous, it shows three different patterns. (B) Joint time-frequency plane representing the overlap of 245 contour plots. In this time-frequency tilling-like pattern representation, each “tile” was obtained from the amplitude envelope and spectral power of the optimized filters at 95% of the energy peak.

**Figure 3:**
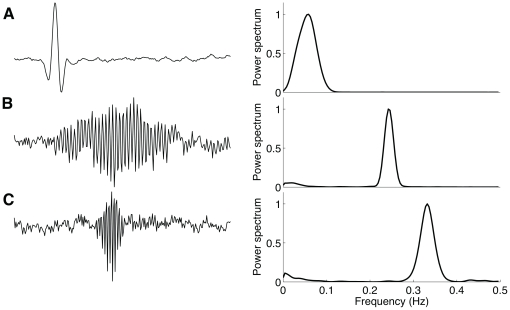
Spectral analysis of the decoding filters. ( *left*) Temporal representation of filters followed by the ( *right*) normalized power spectral whose center frequencies are (A) 0.05 Hz, (B) 0.24 Hz, and (C) 0.33 Hz. Their waveforms remarkably resemble bandpass filters.

The decoding filters emerging from the statistical structures underlying the heartbeat intervals show (**Fig. 2A**) a wide variety of impulse response shapes. The vast majority are time localized, meaning that the analysis window was able to capture the timescale where the statistical regularities of the heartbeat intervals occurred. Despite the observed diversity of sinusoidal oscillations and amplitude envelopes of the filters, the population code has a distinct time-frequency organization (**Fig. 3**). This organization was not clear from the individual analysis of each filter, neither in frequency nor time, but became visible when the entire decoding population was distributed in the joint time and frequency plane (**Fig. 2B**). Moreover, a striking resemblance with the frequency band division of short-term heartbeat intervals emerges. This result is expected, since the encoding filters tend to match the statistical structures underlying the variations of the autonomic cardiac activity. However, nowhere in the algorithm was this structure programmed, i.e. it emerged from the data and the ICA methodology.

Principal component analysis (PCA) is an alternative way to quantify an encoding population. The idea is that the space of responses of an observed system could be replaced by a reduced space of decorrelated and orthogonal functions given by PCA. This fact has lead several authors to attempt to form an optimal representation of an observed signal using PCA functions. The problem is that PCA is strictly a second order decomposition (assumes a Gaussian distribution for the generative model, which is unlikely to be found in biological or natural signals) unable to distinguish between uncorrelatedness and independence. For instance, decoding filters obtained from the same heartbeat ensemble differ appreciably from the ones optimized by ICA. They do not self-organize to explain the modulatory properties of the autonomic system, e.g., sympathetic and vagal tones.

**Figure 4:**
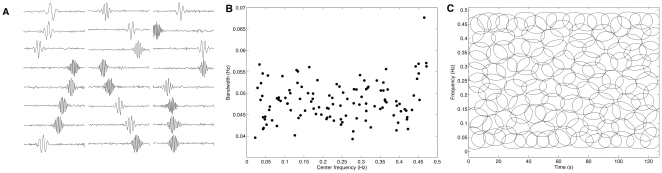
Bias test. (A) Partial representation of a population code composed of (decoding) waveforms learned from ICA using a sparse dataset results in bandpass-like filters. The dataset was drawn from a sub-band modulated signal ensemble constrained to have a 0.05 Hz bandwidth. (B) The center frequency Vs. bandwidth pattern derived from the population code composed of 128 filters illustrates that the learned decoding population have bandwidth centered around 0.05 Hz. (C) Joint time and frequency plane of the decoding population (see **Fig. 2** for details).

Still, can the decoding filters learned by ICA be a result of misestimating the modulatory frequency contributions underlying sympathetic and vagal activity? This is a difficult question to answer in general, but to evaluate the method we created a synthetic dataset composed of sparse structures drawn from a temporal series that was sub-band modulated by a set of bandpass filters. Each filter has the bandwidth constraint to 0.05 Hz, but displaced to cover a frequency range varying from 0.01 until 0.5 Hz. The decoding population emerging from this dataset using ICA have temporal structures that are similar to bandpass-filters (**Fig. 4A**). They have an average bandwidth centered around 0.05 Hz (**Fig. 4B and 4C**). This result supports the accuracy of the estimated decoding population, because the bandwidth of the predicted decoding population matches the design of the bank of filters.

### Time and Frequency Trade-off within the Decoding Population

**Figure 5:**
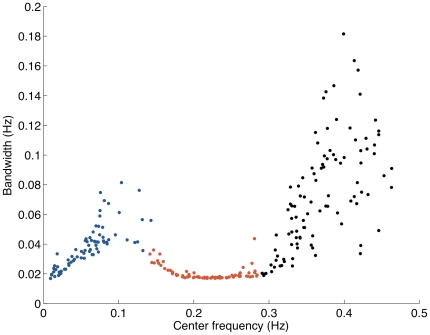
Non-supervised frequency band division. Cluster analysis of the filter center frequency in hertz (horizontal axis) versus the filter bandwidth in hertz for the population code. Each color (blue, red, and black) corresponds to a different cluster (see methods).

**Figure 6:**
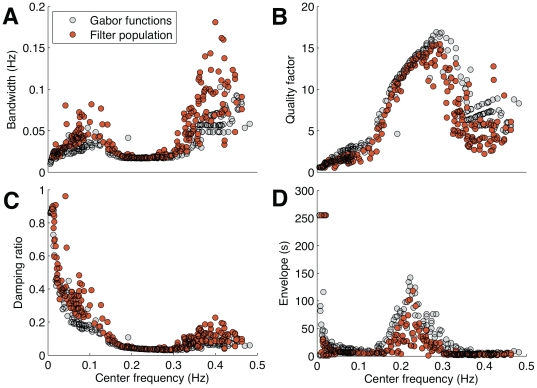
Analysis of the filter behavior. Filter characteristics of the population code (red circle) and its best match with the Gabor function (gray circle) according to Eq. 2. The bandwidth (A) was quantified at -3 dB of the power spectrum maximum amplitude. The quality factor or sharpness (B) represents the ratio between the center frequency and bandwidth of the filters. The damping ratio (C) is a measure based on the quality factor and shows that the filters characteristics have underdamped (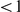) proprieties. The width of each filter temporal envelope (D) was measured at 3 dB below the peak of the energy power using the Hilbert transform.

If the cardiac rhythm accuracy depends on the capacity of the heart to decode sympathetic and parasympathetic stimuli, it should be possible to correlate the time and the frequency distribution of the decoding filters to the cardiac responses. According to previous studies [6], [36], [37], the filtering properties of the auditory system can be characterized using the ratio of center frequency over bandwidth of a population code, called the Q factor. In the cardiac case, we conducted a clustering analysis of the filters' quality factors. We selected the Gaussian Mean Shift (GMS) method because it does not require the selection of the number of clusters, once the kernel bandwidth is chosen from the data (see methods). As shown in **Fig. 5**, we found three different clusters of Q factors that span the 0.01 to 0.5 Hz band, dividing it in regions distinct from the traditional VLF, LF and HF bands. The analysis of the joint decoding population shows that the bandwidth of the filters for VLF and LF increases almost linearly with the center frequency, i.e. a constant Q filter bank that preserves the time resolution (**Fig. 6A**). For HF, the bandwidth is nearly constant for center frequencies ranging from 0.14 to 0.29 Hz, favoring spectral resolution instead of time resolution. And, it increases gradually with the steepest slope between center frequencies located at 0.29 and 0.5 Hz, again preserving the time resolution (see **Fig. 6A**). These results suggest that sympathetic and vagal decoding in the heart are compatible with scale base decompositions such as the wavelet and multirate Fourier transforms. But, why did the heart evolve to use multiscale transform properties to decode sympathetic and vagal contributions in this way? One argument to explain the decoding filter characteristics is the fundamental compromise, captured in the Gabor uncertainty relation [38], between time and frequency resolution. For example, a filter with high-frequency selectivity has a poor time resolution, and vice-versa. Choosing between low- and high-frequency selectivity impinges severe limitations between fast and slow autonomic cardiac regulation and it is one of the obstacles to proper processing in biological systems that are subject to real time response requirements [39]–[41]. Moreover, a fast cardiac response tends to cause a broadening of the filter bandwidth, decreasing the capacity of the system to filter environmental noise at the cost of selectivity. This behavior can be observed by analyzing the quality factor or sharpness of the filters through dividing the center frequency by bandwidth. The ICA decomposition also shows (**Fig. 6B**) that HF have a lower susceptibility to unwanted artifacts than VLF and LF, specifically around 0.14 to 0.29 Hz, where the respiratory sinus arrhythmia (RSA) synchronization is located [42]. This result is consistent with the fact that a sympathetic contribution increases the cardiac rhythm, whereas vagal activity behaves in an opposite way. It is also directly related to the system time response (damping ratio), which is represented by the inverse of the quality factor value multiplied by two. A high-damping ratio means a fast cardiac activity in contrast to low-damping ratio (**Fig. 6C**). Furthermore, filters with broad envelopes are likely to be optimized to process sinusoidal waveforms. Thus, the analysis of the filter envelope patterns suggests that the RSA frequency contributions are happening more frequently. The frequency region where the envelopes have higher values are concentrated between 0.14 and 0.29 Hz (**Fig. 6D**), which is consistent with experimental studies that characterize the respiratory components influencing autonomic cardiac regulation [43].

**Figure 7:**
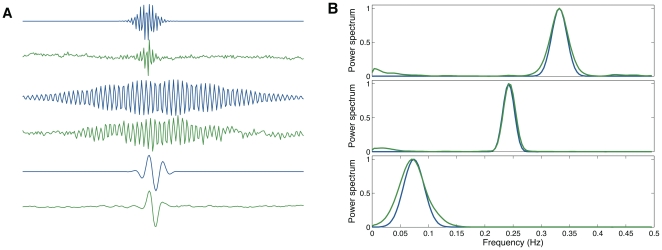
Similarities between the decoding filters (*green*) and Gabor functions (*blue*). (A) Temporal structure. (B) Power spectrum distribution.

As a first approximation, the emerged decoding filters can be modelled by Gabor functions expressed as a Gaussian envelope modulated by a sinusoidal signal as (**Fig. 7**)
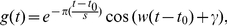
(2)


where the Gabor parameters are defined elsewhere [44]. The matching [45] between the decoding filters and the 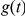 yield a high correlation index (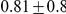; mean 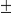 SD, 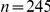) with slightly differences on their filter parameters.

Again, note that no assumption about the filter type, the low/high frequency scaling ratio nor the division into frequency bands are included in our approach. The ICA procedure naturally finds the filters that better adapts to the statistical structure in the heartbeat dataset. If each filter has a preferred frequency range that corresponds to a cardiac response, combining these frequencies would probably result in different cardiac rhythms. Thus, this methodology is appropriate to speculate whether or not sympathetic and parasympathetic contributions are statistically independent. A separate identification of vagal and sympathetic influences could explain why vagal and sympathetic influences are sometimes driven in the same direction.

### Model Response Compared to Physiological Measurements

**Figure 8:**
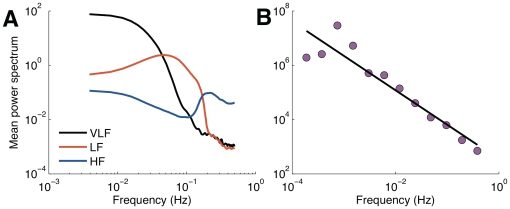
Power law analysis. (A) Power spectrum of the averaged set of decoding population code. (Black) Very-low, (red) low, and (blue) high frequency bands. (B) Binned log-log plot of the filter response (245 filters) to a Gaussian white noise. The straight line represents the power decay with slope 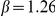.

Despite the well-known sympathovagal balance, one of the inherent properties of heartbeat intervals is that the amplitude spectrum of beat-to-beat variations decays according to the 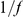 power law [46]. If the average power spectrum of the filter bank decreases linearly with frequency (**Fig. 8A**), one can expect that the decoding filters modulate its input signal to have a 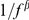 falloff. Thus, could the decoding population itself give rise to long-range correlations close to the ones reported in physiological studies? One way to verify this is to convolve a temporal series drawn from a spectrally white random distribution with the decoding filters. After 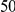 repetitions, the average response of the decoding filters (**Fig. 8B**) to a Gaussian white noise yields a slope 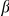 that varies from 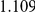 to 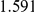 (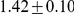, 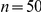). The variability at the low end of the spectrum is expected because of the limited duration of the analysis window (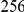 samples) that precludes good estimation of the filters at the low end of the spectrum, again due to Gabor's uncertainty relation.

**Figure 9:**
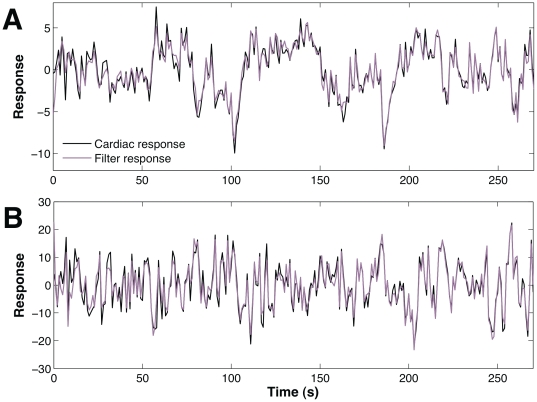
Comparing cardiac response (black line) with filter response (magenta line). The responses are shown in units representing the standard deviation. (A) The sympathetic system response to a stimulus intensity chosen from a continuous signal that was drawn randomly from a Gaussian distribution in contrast to a decoding filter response (SNR = 14.05 dB). (B) The corresponding vagal nerve (PNS) response and its estimated response using a decoding filter (SNR = 12.62 dB). Besides the fast oscillations, the decoding filters yielded a response the tracks fairly well the observed (physiological) cardiac response.

**Figure 10:**
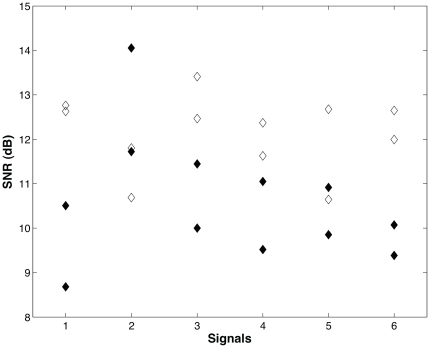
Signal-to-noise ratio between (physiological) cardiac and filter responses for six different time varying stimulus. Response to sympathetic stimulus (⧫) and response to vagal stimulus (◊).

To establish if the estimated filters are indeed a representational form of cardiac population code, one must show that the optimized filters decode an incoming signal similarly to the SA node. Thus, we carried out a comparison based on the responses of the SA node and the decoding filters to a uniform stimulus varying in time. The problem is that, at the moment, is very hard to predict which decoding filter evokes the most similar response to the SA node. One form to circumvent this problem is to find a set of 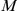 filters that minimizes the error 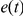 between the responses of the sinoatrial node 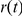 and the decoding filter 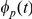 to any given stimuli 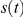 according to [47],
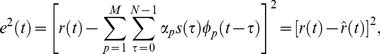
(3)


where 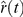 represents the estimated filter response and 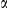 a scaling factor (see methods). We have to include the search for the best scaling factor because the ICA decomposition is blind to scaling. Using a dataset derived from rabbits in a time window 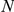 (see methods), the decoding population overall response is smoother then the observed cardiac output. The estimated responses, for both sympathetic (**Fig. 9A**) and vagal (**Fig. 9B**) stimulation follow the expected heart response, but lack the fast oscillations. We also quantify the reliability of the decoding response by measuring the relationship between the cardiac response and the estimated noise 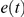 in (3) using the signal-to-noise ratio, SNR(dB) 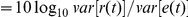, by sliding a time-window length of 270 seconds over 24 heart rate intervals. The SNR varies from 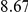 to 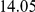 dB ( **Fig. 10**), which correspond to approximately 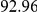 and 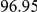 percent of accuracy in psychophysics [48] (see methods).

## Discussion

The statistical encoding and decoding of information in the perceptual system has drawn a lot of attention in recent years [1]–[3], [7]. In the visual system, the ICA methodology is applied to learn efficient codes from natural images and yields spatial filters [4], [8], [49]. In the auditory system, the ICA methodology is used to derive an optimal code for natural sounds in an analysis window (analogous to our work) and provides an explanation to the time and frequency properties of the auditory nerve responses [6]. Our results are relevant to the study of neural information processing systems because they present for the first time an efficient coding principle beyond sensory systems. The ICA methodology is applied to describe functional optimization in the autonomic cardiac response, which is plausible due to the difference in time scales between the neural action potentials and the slow response of the heart muscle. This enables the estimation of filters impulse responses in a linear independent mixture of time filters. The reason we are able to estimate the filters impulse responses in the generative model is due to the assumption that the neuroregulatory messages are sparse (i.e. zero/one signals) modelled mathematically as delta functions, which makes the filter impulse responses statistical independent (if the delta functions do not overlap in time). Therefore, we venture to say that the information theoretic principle of redundancy reduction seems to be appropriate to explain self-organized functional optimization in multiple organs and that an effort should be made to create synergisms between this multidisciplinary knowledge. For instance, if we hypothesize that the nervous system is adjusted to account for the statistical proprieties of the environment in which it is exposed, the efficient coding hypothesis [19], [20] can be extended to the cardiac system whose function is also largely dependent upon the input stimuli. Accordingly, a specialized cell is likely to behave as a feature detector (e.g., a filter) if it only responds to very distinct stimulus [50]. Contextually, cardiac pacemaker cells have been described as a large network of oscillators with different intrinsic frequencies that synchronize (phase-lock) and fire together (beat) [51]. Remarkably, a number of reports [11], [12] raised evidence that the sinoatrial node regulation varies according to specific sympathetic and vagal frequency stimuli. Theoretical studies, on the other hand, have shown that the behavior of heartbeat intervals could be scaled using wavelet-like filters (Gaussian derivatives), but the authors could not explain the basis to the robust temporal structure underlying the cardiac rhythm [52]. We submit that the efficient coding hypothesis explains this robustness. As another example, it is known that cardiac pacemaker cells synchronize their intrinsic frequency to drive the heart rhythm within a limited number of cardiac response levels, but the mechanisms are unclear. Analogous rules can be found in large monopolar cells in the fly's compound eye where receptive fields are known to be created by lateral inhibition [53]. This analogy suggests that the heart may also exploit lateral inhibition to reduce the variability of the responses to certain levels, which can be obtained by minimizing the correlations that probably exist between autonomic stimuli arriving from different pathways similarly to redundant stimuli arriving at sensory neurons.

From the ICA model we conclude that the cardiac system seems to have evolved and be adapted to an unpredictable environment of stimuli [54]. Furthermore, if the decoding filter shapes are adapted to the statistical structure underlying the heartbeat intervals, then one can advance a hypothesis about how the population code in the heart decodes neuroregulatory messages and how their organization can be interpreted in terms of time and frequency selectivity. Our results show that the quality factor varies according to the center frequency of the decoding filters. It suggests that the heart has a mechanism to alter the filter sharpness to regulate the cardiac rhythm. These variations are consistent with the standard frequency band division proposed to analyze the autonomic cardiac fluctuations [9]. Anatomically, the entrainment of the pacemaker cells have some similarities to the injection locking in lasers [39], [55]. It can be interpreted as a mechanism to reduce the noise and amplify the salience of neuroregulatory stimuli, promoting a faithful decoding of the regulatory signal. Our results also shows that VLF and LF decoding filters are likely to process signals with high temporal resolution, which in turn cause susceptibility to noise due to the low quality factor. On the contrary, HF filters have high-frequency selectivity and are more immune to noise. Such time and frequency selectivity agrees with the idea that the responses of the cardiac rhythm are enhanced by noise [56], [57]. This versatility of responses seems to be a reliable option to adjust the cardiac regulation against life threatening conditions, but only when fast cardiac responses are essential for survival. Altogether, these results corroborate the view that the cardiac system is optimized to use the regulatory information in a proper manner to maintain the accuracy of the heartbeat. That is, without introducing redundancy, and preserving energy [58].

A common abstraction in computational perception is the analogy of natural images with edges, where an edge can be represented by a superposition of Gabor-like functions [5], [59]. In an analogous manner, gammatone filters are the corresponding optimal decoders for natural sounds [6], [37], [59]. We have found that the closest equivalent to an optimal decoder for heartbeat intervals is the Gabor wavelet-like function. In theory, if a signal power is equally divided among a set of filters whose organization has a wavelet-like time and frequency resolution, the linear filter bank organization can be adjusted to have the same amount of information at each filter output (channel) resulting in a 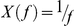 distribution [60], [61]. We have experimentally shown that integrating the decoding filter responses to a flat log-log power resemble an amplitude spectrum that decays with a 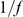 distribution (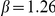, as shown in **Fig. 8B**) for frequencies above 0.005 Hz. This experiment lends support, rather than rules out the hypothesis that heartbeat intervals amplitude spectrum is modulated by a set of filters processing neuroregulatory information. Recall that no attempt was made to select any of the filters to approach the spectral proprieties of heartbeat intervals. This is controlled by the statistics of the data and our selection of the observation interval. The observation interval imposes a limit not only on the maximum number of filters, but also in the maximum length of their impulse response. The accuracy of the filters' impulse response at lower frequencies is compromised by the analysis window (whose size may be truncating their impulse responses), which corroborates our results in **Fig. 8B**. The frequency resolution for our methodology is estimated to be 0.003 Hz. However, longer observation windows may challenge the independence requirement that is the basis of our ICA methodology. It should be interesting to test whether a careful selection of the filters to avoid truncation of impulse responses due to impulses too close to the boundary and window size could result in a more realistic scaling exponents as those observed in healthy subjects (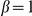). Physiologically, the scale-invariance of the heartbeat intervals should be interpreted as a mechanism that adapts the statistical structure of the input signal to the preferred structure of the encoding mechanisms, just like in the somatosensory neurons [1], [62], [63].

Compared to the present paper, earlier experimental studies [11], [12], [64]–[66] have been able to map the autonomic functions attached to the cardiac system using input-output relationships. Specifically, they characterize the cardiac responses to either invasive vagal or sympathetic stimuli within a reduced dynamic range. However, it is debatable if they reflect natural cardiac states because specialized circulatory mechanisms and feedback systems are often sectioned off to avoid the influence of components other than sympathetic and vagal contributions. Moreover, the transfer analysis based on both sympathetic and vagal stimuli is still challenging due to several unknown factors [11], [65].

In contrast, our work uses directly the statistical structure that can be predicted from the heartbeat intervals to access the autonomic regulation. Clearly, one of the advantages of our work lies on allowing an analysis of autonomic interactions without disturbing the physiology. Indeed, it is remarkable that reducing the redundancy in heartbeat intervals yields features whose characteristics explain several aspects of the cardiac behavior. Nevertheless, the limitations of our noninvasive and computationally simple model are also worthwhile to be described. First, the modeling is functional (generative model) and does not point directly to the physiology. Second, the model lacks feedback connections known to exist from lower to high order processing stages to mediate the control of the cardiac dynamic range. Note that although feed-forward arrangements play an important role to describe cardiac rhythms (such as baroreflex mechanisms and circadian variations), they don't give any information about how the cardiac circuitry self-organize to provide heartbeat control. Third, our analysis is limited to healthy volunteers and does not account for pathophysiological changes. Fourth, even though our results provide an elegant connection between the statistics of heartbeat intervals and the neuronal processing in the heart, the ICA model cannot capture all dynamic patterns. Among them, the magnitude of the transfer function and the shape of the phase curves. Nor can it determine the effect of the interaction between sympathetic and vagal stimulation. Including these characteristics into a cardiac system design could probably increase the accuracy and improve the robustness of the model. Therefore understanding the computational aspects underlying these characteristics is an important step for further research in this area.

The relationship between the known cardiac dynamics and the model hypothesis should be closely analyzed. For instance, previous research on cardiac dynamics has shown that the sympathovagal interactions regulating heart rate display nonlinear behavior [12]. At the surface, the concept of linear filters assumed in our generative model may appear inconsistent with the cardiac dynamics. However, this is not the case because in our model the linear filters switch in time, producing an aggregate response that is compatible with a global nonlinear filter [67] (i.e. the mixture of experts implements exactly a similar methodology). Accordingly, if the nonlinear coding behavior is likely to happen in high-order neural processing connected to the extraction of complex features [68], the filters could also represent some dynamics at higher autonomic levels. It is interesting to note that in our model the switching is actually controlled by the input, so there is no need for a gating network as in the mixture model. But further development of the heart ICA model may complement this gating by feedback from the output, which may bring the feedback loops that are known to exist in the heart from lower to high order processing stages to mediate the control of the cardiac dynamic range. In order to derive our model, we have assumed that short time heartbeat intervals are stationary. But once the filters are learned from the data the overall model can still be applied to long term studies, because the filters are totally controlled by the input stimuli that can vary over longer time scales. This is similar to Gabor wavelets transforms, which are appropriate to unveil dynamic proprieties concealed by non-stationarity [52]. We have also found compelling evidence that the joint response of the filters can approximate the response of the cardiac system. By directly comparing the similarity between the response of the decoding filters and the cardiac system, we aim to directly test whether or not the decoding filters are able to predict the cardiac response. We assume that this process has the same quantitative value as comparing the predicted filter proprieties to the ones estimated physiologically [5], [6], [36].

Cardiac dynamics may vary according to specific physiological functions (such as the thermoregulation and the respiratory rate of each species); therefore, the parameters attributed to heartbeat oscillations could happen at different frequencies when compared to the human physiology. Indeed it has been reported that the high frequency band of rabbits is localized at higher frequencies than humans, whereas very-low and low frequency band limits remain unaltered [69]. Although we have learned the filters from heartbeat intervals derived from humans, the filters were able to yield a response that matches the cardiac response of physiological data obtained from rabbits. Of note, however, the learned filters span their frequency range, so that it covers frequencies up to 0.5 Hz (healthy volunteers). Therefore, the model cannot discern the faster frequencies (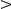 0.5 Hz) that are expected to exist in the physiological measurements obtained from rabbits. Moreover, the objective of the generative model is to evidence properties of the generation mechanisms underlying the heart beat variations, rather than focusing on particular hemodynamic parameters or searching for a specific transformation. This is consistent with filters learned from neural networks optimized to code natural images or natural sounds, whose characteristics resemble the response proprieties of the cells found into the receptive fields in V1 and inner ear [5], [6].

From an anatomical point of view, we suspect that the filters may be located at the SA node, which is the pacemaker structure of the heart. It has been reported that the size of the pacemaker cells localized at the SA node gradually increase from the center to periphery [70]. Where, the cell capacitance (which is proportional to the size of each cell) had a significant correlation with the pacemaker cycle length, meaning that each cell would be tuned to a given frequency, similarly to a band-pass filter that integrates specific information.

Our analysis offers a hypothesis to explain the strategy used by the heart to regulate the cardiac rhythm. That is, by learning a reduced number of mathematical descriptors (filters) according to the efficient coding paradigm, we are able to describe operational point changes of the cardiac regulation that could result in a wide variety of heart rhythms. The challenge, however, is to design computational models that could use the combined filter response to raise insights about the sympathovagal interactions. Advanced models could even be used to simulate several other aspects of the autonomic regulation, such as cardiac gain control and masking effects (inhibitory and excitatory). In more general terms, the fundamental aspects of our study might be appropriate to analyze other neural circuits such as the regulation of glands and smooth muscles, where sympathovagal interactions aim to establish a dynamic equilibrium; and the respiratory control system in which self-tuning adaptive regulation is essential to maintain homeostasis. In conclusion, it seems that efficient coding theory may represent a much broader principle to explain how biological systems process information than its initial application to sensory systems [20].

## Methods

### The Generative Model

Let us consider a bank of 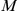 linear FIR filters with impulse response 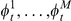 of unknown coefficients and arbitrary length, where the longest filter has a duration 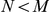. Each filter is excited by a realization of a point process (single delta function 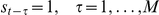 and 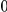 everywhere else in the interval 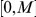) of unknown arrival time 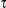 in the interval 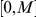 and unknown amplitude. The impulse response of the 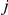-th filter is denoted 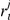 and given by
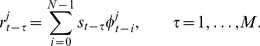
(4)


For simplicity, the convolution sum can be extended to 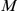. Neither the excitation nor the filter outputs are observable. The measurable quantity is
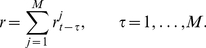
(5)


The signal 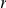 is segmented into at least 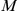 windows of duration 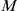. We further assume that the 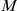 delta functions inputs to the filter bank do not overlap in time during each observation period 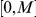, making them statistically independent. Therefore, the impulse response of the filters in the filter bank are statistically independent also (although they very likely will overlap in 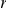). We will also assume that 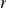 is locally stationary during 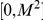.

We claim that this generative model for 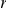 can be framed as an instantaneous mixture of independent filters and that their impulse responses can be estimated from 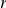 using established algorithms from the field of independent component analysis (ICA). Using matrix notation the ICA problem can be described as follows. The ICA problem (addressed in this paper) focus on coding (by maximizing information of) an observed random matrix 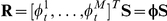, where 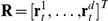 and 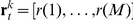. The matrix 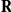 is pre-whitened and has zero-mean, and the matrix 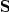 represents a set of vector codes given by latent variables that are statistically independent and non-Gaussian. The matrix 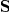 can be obtained as a linear transformation of 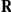, such that 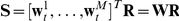, where 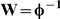. If each vector code can be expressed as 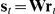 , then it is easy to see that the rows of 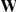 yield finite impulse response (FIR) filters, namely 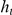, and similarly the columns of 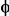 yield FIR filters 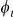 [71].

Our hypotheses have the following implications for the cardiac system. The neural messages impinging into the heart and the filter responses specified by the heart muscle exist at two different time scales: the neural messages occur at a much faster time scale (few milliseconds); while the filters have frequency response in the tenths of Hz, hence the time scale difference between impulses responses and neural messages is at least two orders of magnitude. Therefore, the assumption of delta functions excitation is reasonable, which makes the interpretation of 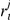 as an impulse response of the unknown filter appropriate. The action potential shape and the sparsity of neural firings in time will also make the assumption of 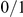, nonoverlapping excitation of the neural filters reasonable.

### The FastICA Algorithm

A detailed description of the FastICA algorithm have been previously reported elsewhere [35]. Briefly, the coding matrix 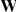 is obtained by a repetitive process of optimization. It uses an approximation of the *negentropy* that maximizes the non-Gaussianity of 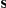 through parallel one-unit iterations and symmetric orthogonalization. In an elementwise operation it can be expressed as [72]


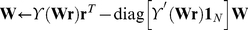
(6)


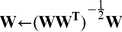
(7)

where 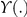 and 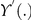 correspond respectively to the first and second derivative of a nonquadratic function herein represented by 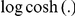. Moreover, the 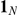 stands for a unitary vector 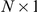. After constraining the matrix 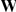 to unit norm, we test the experiment using five different numbers of iterations to investigate the accuracy of the results. That is, the adaptive process was optimized through 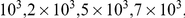 and 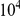 iterations each yielding its own 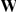 matrix. Across all trials, the matrix 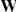 shows no qualitative differences. It is easy to see that the filters (rows of 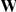) are optimized in a specific way that their waveforms do not depend upon any particular constrain. Their shape is adapted to maximize the information contained on the statistical structure of heartbeat intervals.

### Cluster Analysis

The statistical data analysis of the filter proprerties (center frequency and bandwidth) are assigned into subsets (clusters) using an unsupervised method called Gaussian mean shift [73]. That is, given a dataset 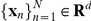, and defining a kernel density estimate
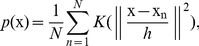
(8)


and assuming that 
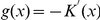
, the previous equation can be rearranged (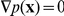) into an iterative process, such that
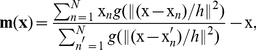
(9)


is denominated mean shift scheme, which represents the difference between the weighted mean and the 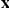. Using a Gaussian kernel 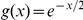, the mean-shift algorithm can be expressed according to:
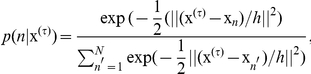


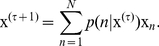
(10)


The cluster bandwidth, 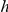, was estimated using a K-nearest neighbor algorithm with 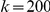. We selected the Gaussian mean-shift algorithm because there is no need to select the number of clusters, which is unknown in our case.

### Datasets

Herein, we used two datasets: heartbeat intervals from normal sinus rhythm and heart rate signals derived from Japanese rabbits. **(i)** The heartbeat intervals database was used to estimate the matrix. The heartbeat data is described in [74] and it is freely available at http://www.physionet.org/physiobank/database/ nsr2db/. Shortly, they consist of records of heartbeat intervals derived from 24 hours of ECG signals sampled at 128 Hz. They were obtained from a total of 59 volunteers, each one of them reporting normal sinus rhythm pattern. The volunteers were composed of 30 men (varying from 28.5 to 76 years old) and 24 women (varying from 58 to 73 years old). These data were then organized into 256-sample segments and filtered by an adaptive process [75] to correct undesired artifacts, such as ectopic beats. It resulted in a total of 22,685 non-overlapping segments that upon whitening process were reduced to 256 segments yielding the random vector. **(ii)** Alternatively, a heart rate database was used to test the capacity of the decoding filters to obtain the cardiac responses. It consists of cardiac responses to a time varying stimulus corresponding to band-limited Gaussian white noise. They are composed of six signals whose input is a stimuli and the output the heart rate sampled at 200 Hz and 10 minutes long. Each signal was resampled at 1 Hz and segmented into intervals of 270 seconds, resulting in 24 segments corresponding to the cardiac response to sympathetic and vagal stimulation. The details about the surgical and experimental procedures where previously described elsewhere [65]. And, they are briefly summarized in the next subsections.

### Surgery

All the following animal procedures are in agreement with guiding principles of the Physiological Society of Japan. Herein intravenous injections (2 ml/kg) containing urethan (250 mg/ml) and 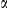-chloralose (40 mg/ml) were used to anesthetize eight white Japanese rabbits (2.3–3.3 Kg), while they were mechanically ventilated using oxygen-enriched room air. During surgical procedure and stimulation, additional anesthesia doses (0.5 ml/kg) were injected, when necessary, to ensure a proper anesthesia level. For the purpose of monitor aortic pressure, a catheter (via femoral artery) was used. The effects of arterial baroreceptors flexes were removed by bilaterally cutting the carotid sinus and aortic depressor nerves using a midline cervical incision. Moreover, feedback effects arriving from the cardiopulmonary region were removed by sectioning the vagal nerved located at the neck. For sympathetic and vagal nerve stimulation, bipolar platinum electrodes were implanted: one pair at the stellate ganglia (after midline thoracotomy and sectioning the sympathetic nerves) and one pair at the cardiac end of the right vagal nerve. To avoid desiccation and guarantee insulation, a mixture of paraffin and white petroleum (Vaseline) was used to soak both nerves and electrodes. After maintaining body temperature constant (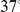
C, using heating pad), cardiac recordings were obtained from a pair of (stainless steel) electrodes (implanted in the right atrium) connected to a cardiotachometer (model N4778, NEC Sanei, Tokyo, Japan) to measure the instantaneous heart rate. The cardiotachometer locate and mark the time positions of the heartbeat events 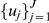 of the heart to compute the heart rate. Defining a time series composed of 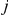 time differences between two consecutive heartbeat events in seconds as 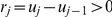 , the (instantaneous indexes of) heart rate is expressed in beats/min as 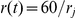.

### Stimulus and Data Recording

The nerve stimuli is comprised of a frequency-modulated signal (frequency stimuli varies every second) drawn from a band-limited Gaussian white noise, whose amplitude varies in time at each 2 ms. Both sympathetic and vagal power spectrum nerve stimuli vary slightly until reaches 0.5 Hz and decays gradually to 1/10 around 0.8 Hz, reaching noise levels as it approaches 1 Hz. The amplitude of sympathetic (1.8–3.8 V) and vagal (4.2–6.2 V) nerve stimuli are, respectively, adjusted to yield a heart rate increase and decrease around 50 beats/min (at frequency stimuli of 5 Hz). Stimuli and yielded instantaneous heart rate response were sampled and recorded at 200 samples per second with a 12-bit resolution (NEC PC-98, Tokyo).

### Decoding

To translate the filters learned from the human experiment to the decoding methodology, we convolved a known continuous signal (used in the physiological experiment with rabbits) resampled at 1 Hz with interpolated filters at 1 Hz to estimate the instantaneous heart rate [as described in (3)]. Because the neural network (based on ICA) used to maximize information is blind to scaling, a scaling factor [as shown in (3)] is introduced to translate the response of the interpolated filters to heart rate signals. That is, taking into account that the filters are expected to span a set of independent basis, the scaling can be solved by projecting the heart rate signal onto the response of the filters.

### Time and Frequency Analysis

The joint time and frequency plane represents the overlapping of contour plots. Each one estimated through a type II Cohen Class using a spectrogram kernel [76] whose input is the Hilbert transform of the decoding filters 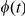. The Hilbert transform 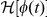 is represented by the imaginary part of the analytical signal 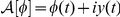, where 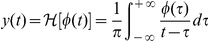. The envelope is then expressed as 
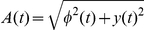
. Some of the estimated filters are not well localized in frequency. Those filters whose spectral power is not concentrated in one peak were excluded. From the total of 256 filters, three in VLF and eight in LF were omitted from the analysis.

### Signal-to-noise and Accuracy

The percentage of reconstruction accuracy 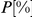 is measured based on SNR in a psychophysics context as
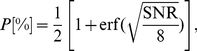
(11)


where 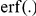 represents the error function.
